# From surface to depth: Using deep learning to predict striatal fMRI reward signaling from EEG

**DOI:** 10.1162/IMAG.a.1160

**Published:** 2026-03-09

**Authors:** Nadine Herzog, Luka Vähäsarja, Pia Reinfeld, Nico Scherf, Simon M. Hofmann, Christina Andreou, Arno Villringer, Christoph Mulert

**Affiliations:** Department of Neurology, Max Planck Institute for Human Cognitive & Brain Sciences, Leipzig, Germany; Center for Psychiatry, Justus Liebig University, Giessen, Germany; Department of Medicine, University of Helsinki, Helsinki, Finland; University of Applied Sciences Jena, Jena, Germany; IMPRS CoNI, Max Planck Research School on Cognitive NeuroImaging, Leipzig, Germany; Neural Data Science and Statistical Computing Group, Max Planck Institute for Human Cognitive and Brain Sciences, Leipzig, Germany; Center for Scalable Data Analytics and Artificial Intelligence (ScaDS.AI), Dresden/Leipzig, Germany; Translational Psychiatry Unit, Department of Psychiatry and Psychotherapy, University Hospital Schleswig-Holstein, Lübeck, Germany

**Keywords:** EEG–fMRI, deep learning, reward processing, ventral striatum, neurofeedback

## Abstract

Reward processing is critical for motivation, learning, and decision making. It involves a network centered on the fronto-striatal circuit, with the ventral striatum (VS) playing a pivotal role. While functional magnetic resonance imaging (fMRI) has been instrumental in mapping subcortical VS reward signals, its cost and limited accessibility hinder broader clinical applications. In this study, we adapted a convolutional autoencoder deep learning (DL) model to reconstruct VS blood-oxygen-level-dependent (BOLD) activity from task-based electroencephalography (EEG) data, both recorded during a two-choice gambling task known to elicit reward-related activation in the VS. The model was trained on consecutive EEG–fMRI data from 19 healthy participants, allowing it to identify patterns that generalize across individuals. Results show that the DL model significantly outperforms linear baseline models in predicting VS activity: across leave-one-out folds, the mean correlation between the DL-derived and the ground truth VS BOLD signal was $\bar{r}=0.323$, compared with $\bar{r}=0.213$ for the linear model. Further validation confirmed that the DL-derived signal is anatomically specific to the VS and other reward-related areas, and that it is modulated by reward conditions, indicating its functional validity. EEG feature analyses revealed theta to beta frequency band involvement, particularly in right centroparietal and temporal, as well as frontal electrodes. Although the model’s generalization performance was modest, these findings demonstrate the feasibility of decoding subcortical reward-related signals from surface EEG using interpretable deep learning models. This work contributes to the foundation for EEG-based neurofeedback systems aimed at modulating subcortical reward circuits, with potential clinical applications for disorders characterized by impairments in these circuits. Future improvements in model generalization may be achieved by training on larger and more diverse datasets.

## 1 Introduction

Reward processing is a fundamental aspect of human cognition and behavior, playing a central role in motivation, learning, and decision making (Berridge & Robinson, 2003). Broadly, rewards are stimuli that elicit approach behaviors, produce subjective pleasure, and reinforce associated cues and actions (Schultz, 2006, 2015). The regulation of psychological and behavioral responses to rewarding stimuli is coordinated by a collection of cortical and subcortical structures forming the brain’s reward circuit (Haber & Knutson, 2010). At the core of this circuit is the mesolimbic pathway, which includes structures such as the ventral striatum and the dopaminergic midbrain. Upon receiving a rewarding stimulus, the ventral tegmental area releases dopamine into the ventral striatum, generating a signal of pleasure and thus reinforcing the behavior that led to the reward. Crucially, dysfunctions in dopamine signaling are associated with psychiatric symptoms of altered reward processing, such as addiction (Solinas et al., 2019; Wise & Robble, 2020), depression (Admon & Pizzagalli, 2015; Pizzagalli, 2014), apathy (Kirschner et al., 2020), or other conditions such as obesity (García-García et al., 2014; Herzog et al., 2024a; Waltmann et al., 2024). Thus, understanding reward processing is critical for developing effective therapeutic interventions.

Traditionally, functional magnetic resonance imaging (fMRI) has been the primary tool for examining neural substrates of reward, particularly in subcortical areas such as the ventral striatum (Haber & Knutson, 2010; Rosen & Savoy, 2012; Sescousse et al., 2013). However, due to its high cost and lack of mobility, fMRI has restricted broader applications, particularly for clinical purposes. Electroencephalography (EEG) could prove valuable here, as it is non-invasive, cheaper, and more readily accessible. However, scalp EEG signals primarily reflect cortical activity (Amzica & Lopes da Silva, 2011), making it difficult to capture deeper brain structures such as the ventral striatum. This is partly due to the fact that, unlike cortical neurons, ventral striatal neurons are not oriented perpendicularly to the skull surface (Haber, 2011), so their electrical signals do not sum effectively. Additionally, the signal must traverse several brain regions before reaching the scalp, becoming superimposed with adjacent signals and thus further attenuated (Baillet, 2017).

Nevertheless, recent research has demonstrated that, in principle, deep brain activity can be inferred from scalp EEG using machine learning (ML) techniques. Particularly noteworthy here are the studies by Meir-Hasson et al. (2014, 2016) and Singer et al. (2023). In their studies, Meir-Hasson, Singer, and colleagues used ML to generate EEG-based models of activity in the amygdala and ventral striatum, respectively, using simultaneous EEG–fMRI recordings. These models produced EEG-derived time series that significantly correlated with the corresponding fMRI time series extracted directly from the respective area. Singer et al. (2023), for instance, reported a mean correlation of r = 0.26 for the EEG-derived model of ventral striatal activity. Both models also demonstrated high anatomical specificity, as shown through fMRI regression analyses. Moreover, the ML-derived amygdala model has shown clinical relevance in neurofeedback interventions, yielding significant symptom improvements in individuals with post-traumatic stress disorder (Fruchtman-Steinbok et al., 2021). Additionally, a pilot study published only recently demonstrates the practical applicability of Singer et al.’s striatal EEG model for the treatment of depression (Amital et al., 2025). These findings underscore the feasibility of using ML models to extract meaningful signals in the EEG from deep brain structures.

In their studies, Meir-Hasson, Singer, and colleagues utilized ML approaches that are based on simple linear regression models. However, given that—as described above—the potential scalp signal likely comprises numerous dynamic and interwoven components, such linear methods may be inadequate for modeling the complex, nonlinear neural dynamics from deep brain regions such as the ventral striatum. Deep learning (DL)-based ML methods offer a crucial advantage here, as they are capable of capturing possible non-linear relationships between EEG and fMRI signals. The superiority of DL-based over linear ML methods for detecting deep brain activity in EEG has already been demonstrated in studies by Kovalev et al. (2022) and Li et al. (2024). In their work, Kovalev et al. (2022) trained a subject-specific convolutional autoencoder model to reconstruct fMRI activity in the basal ganglia from simultaneously acquired EEG data. Using this DL approach, they achieved a mean correlation of r = 0.42 for the nucleus accumbens (i.e., ventral striatum).

In the present work, we built upon this publicly available DL model developed by Kovalev et al. (2022) to further improve EEG-based detection of reward signals in the ventral striatum. Specifically, we adapted the model—which was originally trained on resting-state data—to task-based data collected during a two-choice gambling reward task (Andreou et al., 2017; Gehring & Willoughby, 2002). Importantly, this paradigm has extensively been validated as a robust method for eliciting reward-related activation in the VS (e.g., Andreou et al., 2017; Camara et al., 2009; Riba et al., 2008). Compared with resting-state data, such targeted task-based data have a higher signal-to-noise ratio, which facilitates more reliable and efficient learning of neural signal representations. In addition, we also employ a different training strategy. Rather than training separate models for each participant, as was done in Kovalev et al. (2022), we train a single group-level model across subjects. This approach aims to identify generalizable EEG–fMRI relationships that can transfer to new, unseen individuals—a critical requirement for clinical applications where individual fMRI calibration is often impractical.

The data for this study were originally collected by Andreou et al. (2017) as part of a different investigation. They comprise simultaneous EEG–fMRI recordings from 22 healthy male participants performing the two-choice gambling task. We applied Kovalev et al.’s DL algorithm to this task-based data, aiming to identify the optimal combination of electrodes and frequency bands that best reconstructs a time series that closely resembles the actual fMRI blood-oxygen-level-dependent (BOLD) signal extracted from the ventral striatum. We then tested the anatomical specificity and reward sensitivity of the DL-derived time series to verify that it accurately reflects reward-dependent neural activity in the ventral striatum.

By combining task-based data with deep learning approaches, we anticipate higher average correlation coefficient than those reported in Kovalev et al. (2022; *r*_nucl.accumbens_ = 0.42) and Singer et al. (2023; *r*_ventral striatum_ = 0.26). Furthermore, regarding anatomical specificity, we expect our DL-derived time series to be predominantly associated with BOLD activation in, specifically, the VS. However, as the VS forms part of a reward circuit (Haber & Knutson, 2010), we expect the DL-derived VS signal to also correlate with additional interrelated regions of the reward network, such as the ventral medial prefrontal cortex (vMPFC) or the anterior cingulate area (ACC). In terms of functional validity, we expect the DL-derived time series to be modulated by reward conditions, that is higher betas for win compared with loss events. Lastly, we expect the DL-derived time series to correlate stronger with the BOLD signal from the VS than with the BOLD from non-reward-related control areas, indicating that the model captures VS-reward-specific activity rather than general BOLD dynamics.

Our work extends prior EEG–fMRI decoding approaches thereby contributing significantly to the development of new methods for constructing striatal BOLD activity from low-cost, wearable scalp EEG recordings. We envision this technology to improve monitoring of striatal reward processing remotely using EEG alone, reducing the reliance on fMRI. Moreover, our model could be integrated into novel neurofeedback interventions targeting psychiatric conditions characterized by impaired reward processing.

## 2 Methods

### 2.1 Participants

The data used in this study was acquired in the context of a study conducted by Andreou et al. (2017). The study was conducted in accordance with the Declaration of Helsinki and was approved by the ethics committee of the Medical Council of Hamburg. All the participants provided written informed consent. Initially 22 participants were recruited among students of the University of Hamburg. Exclusion criteria were lifetime psychotic, bipolar or substance-use disorders, depressive or anxiety disorders in the past year, neurological or major somatic illnesses, and psychotropic or any other medication known to affect cognitive functions. All the participants were nonsmokers and had normal or corrected-to-normal vision. Three subjects were excluded from the analyses (see Andreou et al., 2017). Thus, the data provided to us were from 19 healthy male individuals (age 23.67 ± 3.2 years).

### 2.2 Gambling task

**Fig. 1.**
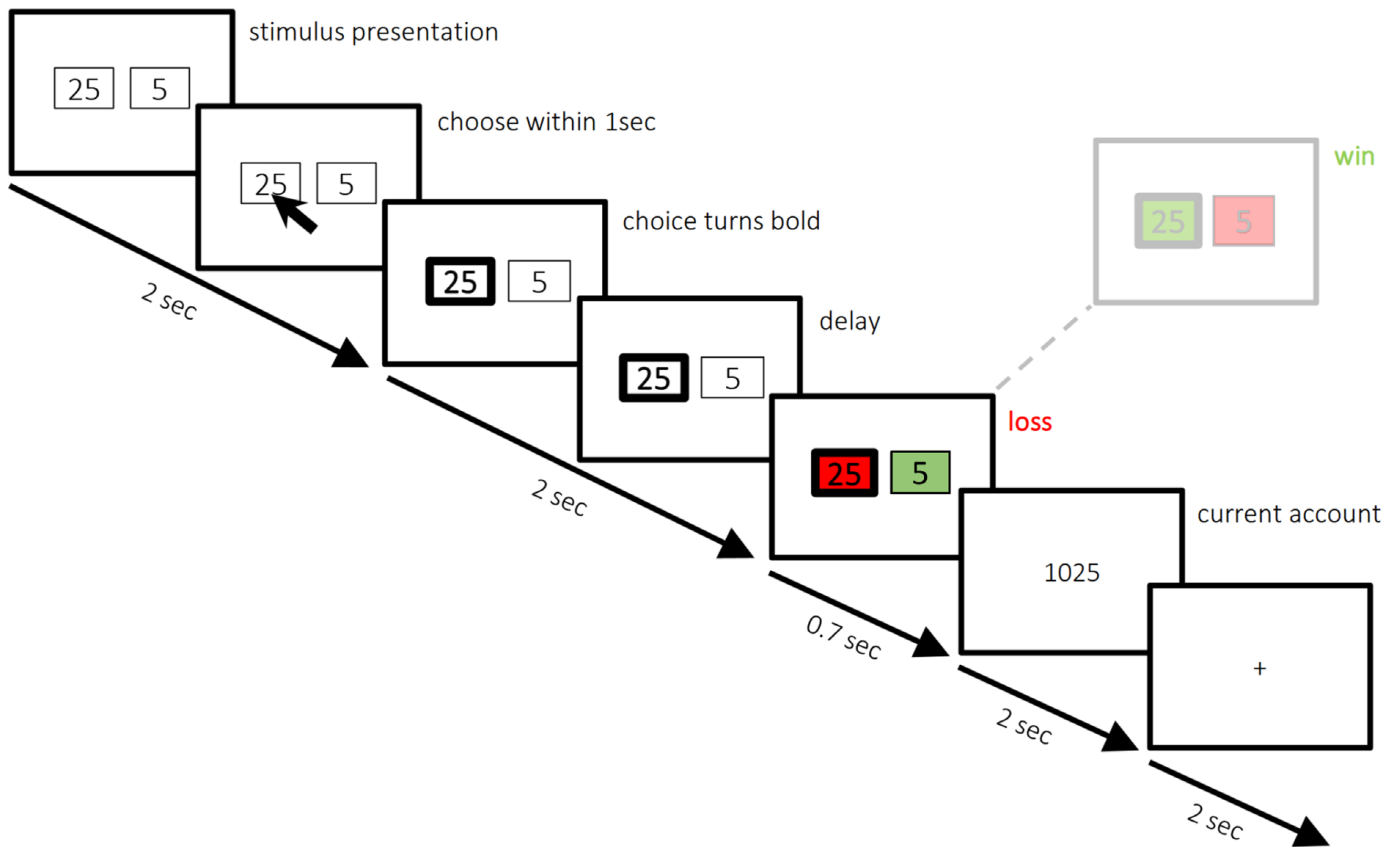
Two-choice gambling task. On each trial, participants were shown two numbers (5 and 25) in a randomized left–right configuration and had 1 second to select one option via button press. Two seconds after trial onset, the selected number was highlighted (bold). Feedback was presented after another 2-second delay, during which the highlighted number either turned green (indicating a gain) or red (indicating a loss). After that, the updated cumulative account score was shown, which was followed by a 2-second fixation period. Participants completed 4 runs of 100 trials and were instructed to maximize their score by freely choosing between high- and low-risk options.

Participants completed a computerized two-choice gambling task, based on the paradigm by Gehring and Willoughby (2002; Fig. 1). Stimuli were presented using Presentation software (version 17) on a computer located in a control room isolated from the MRI scanner. The task comprised 4 runs of 100 trials each. At the start of each trial, two numbers (25 and 5) were displayed on the screen in a randomized left–right arrangement. Participants had 1 second to select one of the two options via button press. If no response was made within this time window, the trial was excluded. Two seconds after trial onset, the chosen number was highlighted in bold. Following another 2-second delay, feedback was provided: one of the two numbers turned green and the other red, indicating whether the chosen value was added to (green) or deducted from (red) the participant’s score. The trial concluded with a 2-second display of the updated score, and each new trial was preceded by a 2-second fixation cross. Participants received standardized instructions and practiced the task beforehand. They were told their goal was to maximize their score, that win and loss outcomes were equally probable, and that they were free to choose between the high- and low-risk options on each trial. Each participant was compensated with 30€ for their participation.

### 2.3 Data acquisition

EEG data were collected simultaneously with fMRI using BrainVision Recorder (v1.10, Brain Products, Munich, Germany) and MR-compatible ACamplifiers (BrainAmp MRplus, Brain Products). The EEG-cap (BrainCapMR 64, Brain Products) featured 62 passive sintered silver/silver chloride electrodes positioned according to a modified 10/10 system, with FCz as the reference and AFz as the ground. Additionally, an EOG electrode was placed below the left eye to account for eye movements and an ECG electrode on the chest provided data for cardioballistographic correction. The ribbon cable linking the electrodes to the amplifiers was secured using sandbags on foam cushions to minimize artifacts from scanner vibrations, and skin–electrode impedance was maintained below 10 kΩ. Data were sampled at 5,000 Hz with a resolution of 0.5 μV. FMRI image acquisition was conducted on a 3-Tesla MRI scanner (Magnetom Trio, Siemens, Munich, Germany) with a 12-channel head coil. Functional imaging employed a standard gradient echo-planar imaging (EPI) T2*-sensitive sequence, capturing 25 slices per volume. Each run produced 530 volumes (TR = 2 s; TE = 25 ms; FOV = 216 mm; matrix size = 108 × 108; continuous slice acquisition; 3 mm slice thickness, with a 1 mm gap between slices). To reduce high-frequency EEG artifacts, the scanner’s vacuum pump was turned off during data collection. Moreover, for each subject, a high-resolution T1-weighted anatomical scan (MPRAGE, 1 × 1 × 1 mm voxel size) was acquired with the same positioning as the EP images.

### 2.4 Data preprocessing

The EEG data were cleaned from the gradient artifacts (Goltz et al., 2015; Nierhaus et al., 2013) and from ballistocardiographic artifacts (using a combination of QRS detection and average artifact subtraction method; Allen et al., 2000). Additionally, ICA was applied to manually identify and remove any remaining artifact components. The data were then downsampled to 1,000 Hz, and a broadband filter with a 0.5–45 Hz passband was applied to remove extraneous noise and low-frequency drifts. The multichannel EEG data were re-referenced to a common average montage.

The fMRI data were processed as described in Andreou et al. (2017) using standard procedures available in Statistical Parameter Mapping (SPM12; Wellcome Department of Imaging Neuroscience, London, UK, www.fil.ion.ucl.ac.uk/spm/). The initial five fMRI volumes were discarded to allow for scanner stabilization. Preprocessing steps included slice timing correction, realignment, normalization to the Montreal Neurological Institute standard space, and spatial smoothing using an 8 mm Gaussian kernel. BOLD responses were analyzed with general linear models. For first-level analyses, the following conditions were modeled as regressors following convolution with a canonical hemodynamic response function: (i) the four win/loss conditions (max gain, max loss, min gain, min loss), (ii) initial stimulus presentation, (iii) motor response, (iv) anticipation phase, and (v) account balance display. Additionally, six motion parameters were included as regressors of no interest.

**Fig. 2.**
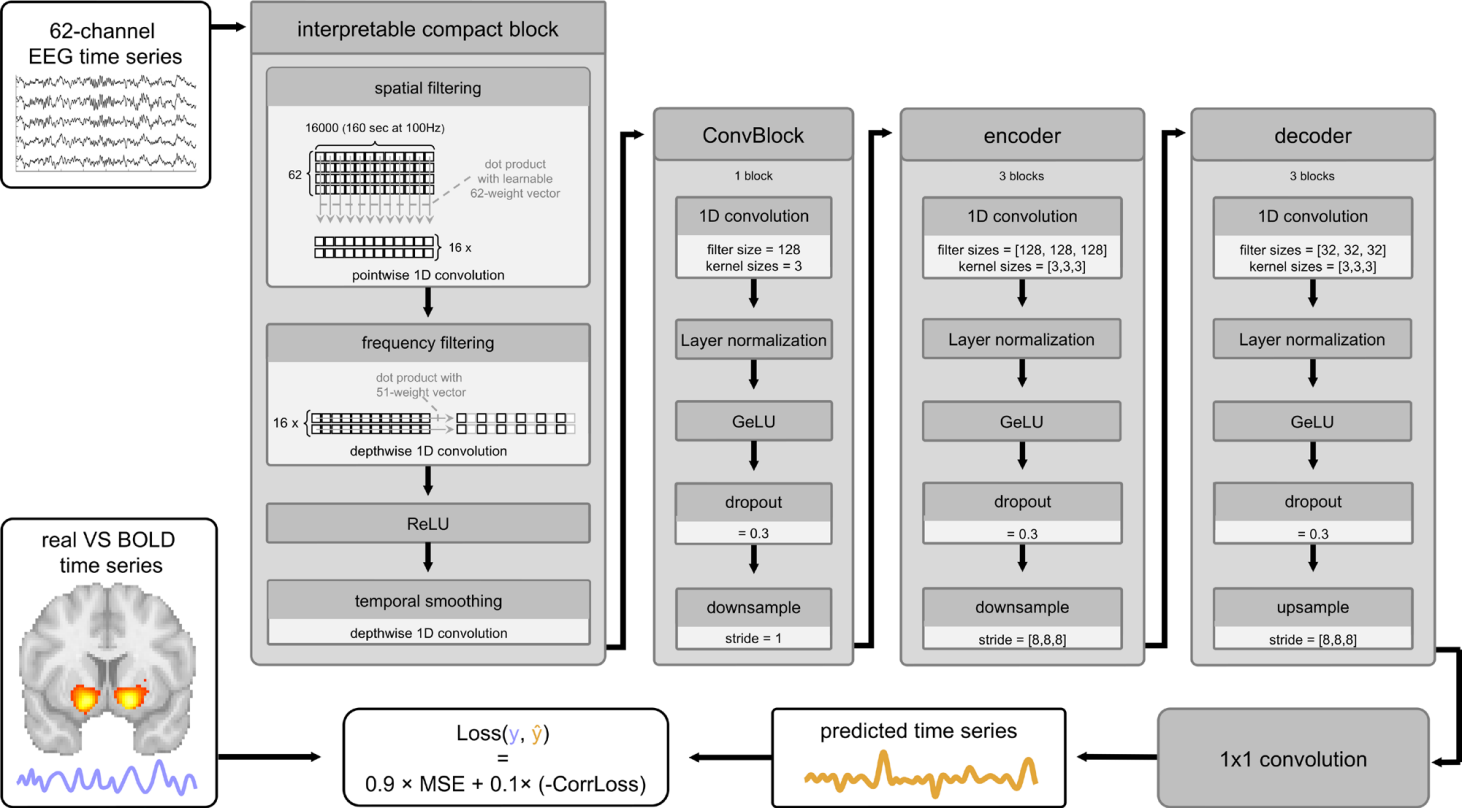
Model architecture for interpretable EEG-to-BOLD mapping, adapted from Kovalev et al. (2022). Preprocessed multichannel EEG data are first processed by the interpretable compact block, which performs spatial filtering via pointwise 1D convolutions, followed by frequency filtering using depth-wise temporal convolutions. ReLU activation and a subsequent temporal smoothing layer further refine the signal. The output is then passed through a convolutional projection block to unify features across branches. This is followed by an encoder–decoder structure, each composed of three layers with 1D convolutions, normalization, GeLU activation, dropout, and down- or upsampling, respectively. A final 1 × 1 convolution projects the decoder output to a single-channel prediction, yielding the model’s final prediction (orange signal). This prediction is then used for loss computation, where it is compared with the true VS BOLD signal (light-blue signal at the lower left).

EEG and fMRI data were temporally aligned by trimming the EEG data to the interval between the MRI trigger immediately preceding the first task stimulus and the trigger following the last stimulus. To verify the alignment, we checked that the number of fMRI volumes corresponded to the number of MRI triggers in the trimmed EEG. For fMRI, time series data were extracted from the ventral striatum (VS) region of interest (ROI) as the average activation across all voxels within the ROI. VS ROI was defined as the area where the win–loss contrast showed the highest significance at the group level (see brain image bottom left in Fig. 2) and, consequently, the highest signal-to-noise ratio. Both EEG and fMRI signals were z-scored. The preprocessed EEG data were down sampled, and the ventral striatum BOLD time series were interpolated to a common sampling rate of 100 Hz. To account for the hemodynamic response delay, the EEG data were shifted by 6 seconds—the delay shown to be optimal in Kovalev et al. (2022) and Singer et al. (2023).

### 2.5 Deep learning model

#### 2.5.1 Model architecture

The model employed in this study, BEIRA (BOLD from EEG Interpretable Regression Autoencoder), was originally developed and validated by Kovalev et al. (2022). BEIRA is a convolutional autoencoder specifically designed for interpretable analysis of EEG data (Fig. 2). EEG (and fMRI) time series data first pass through an interpretable compact block that performs spatial and frequency filtering, extracting spatially and spectrally specific EEG features. Following Kovalev et al., we use 16 branches, each acting as a distinct processing stream, potentially corresponding to a unique neuronal population or spatial/frequency component in the EEG data. Each branch first performs spatial filtering by computing, at each time point, the scalar product between the 62-channel EEG input and a learnable 62-dimensional weight vector. This is implemented as a pointwise 1D convolution layer. The output is a univariate time series representing a spatially filtered signal. This signal is then passed through a depth-wise 1D convolution across the temporal dimension, using a kernel of length 51, to extract frequency-specific features. After that an ReLU activation is applied, followed by temporal smoothing via another 1D convolution with the same kernel length (51). The output of this compact block can be interpreted as a time series representing the power of activity from a spatially localized and frequency-specific neuronal source. Following the compact block, the output from all 16 branches is processed by a shared 1D convolutional layer with a fixed kernel size of 3 and a stride of 1. This layer serves to transform and project the branch outputs into a format suitable for subsequent processing by the encoder–decoder structure. The transformed signals are then passed through the encoder, which consists of three convolutional blocks. Each block applies a 1D convolution with 128 channels and a kernel size of 3, followed by normalization, a GeLU activation function, dropout (with a rate of 0.3), and max pooling for downsampling (stride in each layer = 8). Following the encoder, the data enter the decoder. In the original implementation by Kovalev et al. (2022), the decoder mirrors the encoder’s structure in reverse. Since in our adaptation the filter, kernel, and stride sizes are identical in each layer, there effectively is no reversal. A final linear upsampling layer restores temporal resolution, and the number of channels is reduced by a factor of 4 to lower computational cost. A final 1 × 1 convolution produces the single-channel model prediction.

#### 2.5.2 Model training

We train the model to reconstruct the VSs BOLD signal by solving signal-to-signal translation tasks on consecutive data from all 19 subjects. Following the approach from Singer et al. (2023), we employed a k-fold cross-validation procedure in which each fold held out one run for testing while training on all remaining runs across subjects. Training the model on all available runs across participants served two purposes: it maximized the training dataset size and accounted for potential inter-run variability (also see Meir-Hasson et al., 2016). Due to high computational costs, we implemented 20 random folds rather than an exhaustive leave-one-run-out cross-validation.

To form individual i−th training sample pairs {X_i_, y_i_}, we extract a 160s long window of data formed at randomly selected starting sample index t_i_ as

$${\text{X}}_{\text{i}}\;=\text{X}\left[\text{e}1:\text{e}62,{\text{t}}_{\text{i}}\;:{\text{t}}_{\text{i}}+160\times \text{Fs}\right]{\text{and y}}_{\text{i}}\;=\text{y}\left[{\text{t}}_{\text{i}}\;:\;{\text{t}}_{\text{i}}\;+160\times \text{Fs}\right],$$

where Fs is sampling rate and equals 100 Hz. The model was trained to implement the following transformation: **X** → **y**, where **X** is a 62 × 16,000 matrix of the 62 channel EEG over the 160 seconds long window and **y** is the vector of the corresponding time segment of the VS BOLD signal.

The loss function was defined as a weighted combination of mean squared error (MSE) and correlation loss (CorrLoss) between the DL-derived time series and true BOLD signal. We negated the correlation term to ensure the overall loss function decreases during training:

$$\text{Loss(y},\hat{\text{y}}\text{)}={\text{weight}}_{1}\times \text{MSE}+{\text{weight}}_{2}\times (-\text{CorrLoss}),$$

with weight_1_ = 0.9 and weight_2_ = 0.1.

Training was initialized using an AdamW (Loshchilov & Hutter, 2017) optimizer with a learning rate of 3 × 10^-5^. To stabilize training and improve convergence, we employed a cosine annealing learning rate scheduler (Loshchilov & Hutter, 2016). Specifically, we used PyTorch’s CosineAnnealingLR implementation with a T_max of 50 epochs. We further used a weight decay of 3 × 10^-4^ and a batch size of 32. For fine-tuning the model, we varied these hyperparameters using Optuna (Akiba et al., 2019), an automated hyperparameter optimization framework. To prevent overfitting, we implemented early stopping, that is, training was halted as soon as the loss for the validation subject began to increase while training loss continued to decrease for 15 consecutive epochs. PyTorch was used to implement the training of our models.

### 2.6 Model evaluation, functional validation, and statistical analyses

The model’s performance was assessed by calculating a Pearson correlation between the DL-derived and true VS BOLD time series for each held-out dataset. To determine whether this correlation was statistically significant rather than spurious, we assessed the likelihood of the obtained correlation by determining its percentile location within a null distribution. The null distribution was constructed using a phase-randomization permutation approach (Lerner et al., 2011). Specifically, we repeated the performance calculation 1,000 times, with the critical modification that the VS BOLD signal was phase randomized before computing each correlation. The empirical correlation value was then compared against this distribution of permuted values.

To evaluate the anatomical sensitivity of the DL-derived signal, we further conducted a whole-brain random-effects general linear model analysis on the fMRI data for each dataset, using the DL-derived time series as a regressor. As mentioned above, on the first level, we also modeled all other task events, such as button presses, anticipation, and stimulus presentation. Furthermore, the six head-motion realignment parameters were included to regress out motion-related and other non-neural sources of variance. The resulting regression coefficients for the DL-derived time series regressor per dataset were then submitted to a second-level group analysis using a one-sample t-test in SPM12. We controlled for multiple comparisons by applying family-wise error rate (FWE) correction (Friston et al., 1996). To reduce the likelihood of detecting spurious activations, only clusters consisting of five or more contiguous voxels were considered in the final thresholded statistical maps. The functional validity of the DL-derived time series—that is, whether it is modulated by win versus loss events—was evaluated by fitting a linear model in which the DL time series regressor was modeled alongside the win and loss event regressors, respectively. This was implemented using the fitlm() function in MATLAB (R2024b). For each fold dataset, we then extracted the respective estimate per model to get at the beta values for win versus loss events. To assess the statistical significance of the difference between win- and loss-related beta values, we performed a paired t-test using MATLAB’s t-test() function. Furthermore, to test whether the model captures reward-specific activity rather than general BOLD dynamics, we also correlated the DL-derived time series with BOLD signals stemming from three control regions: the visual cortex, the subthalamic nucleus, and the premotor cortex. Lastly, as a baseline comparison, we also trained simple regularized linear models, following the approach described in Kovalev et al. (2022). To ensure a fair comparison, these linear models were trained on the same permutation folds as used for the DL model. We then assessed whether the DL model outperformed the linear baseline by comparing their correlation coefficients using paired t-tests.

## 3 Results

### 3.1 Model performance and prediction accuracy

**Fig. 3.**
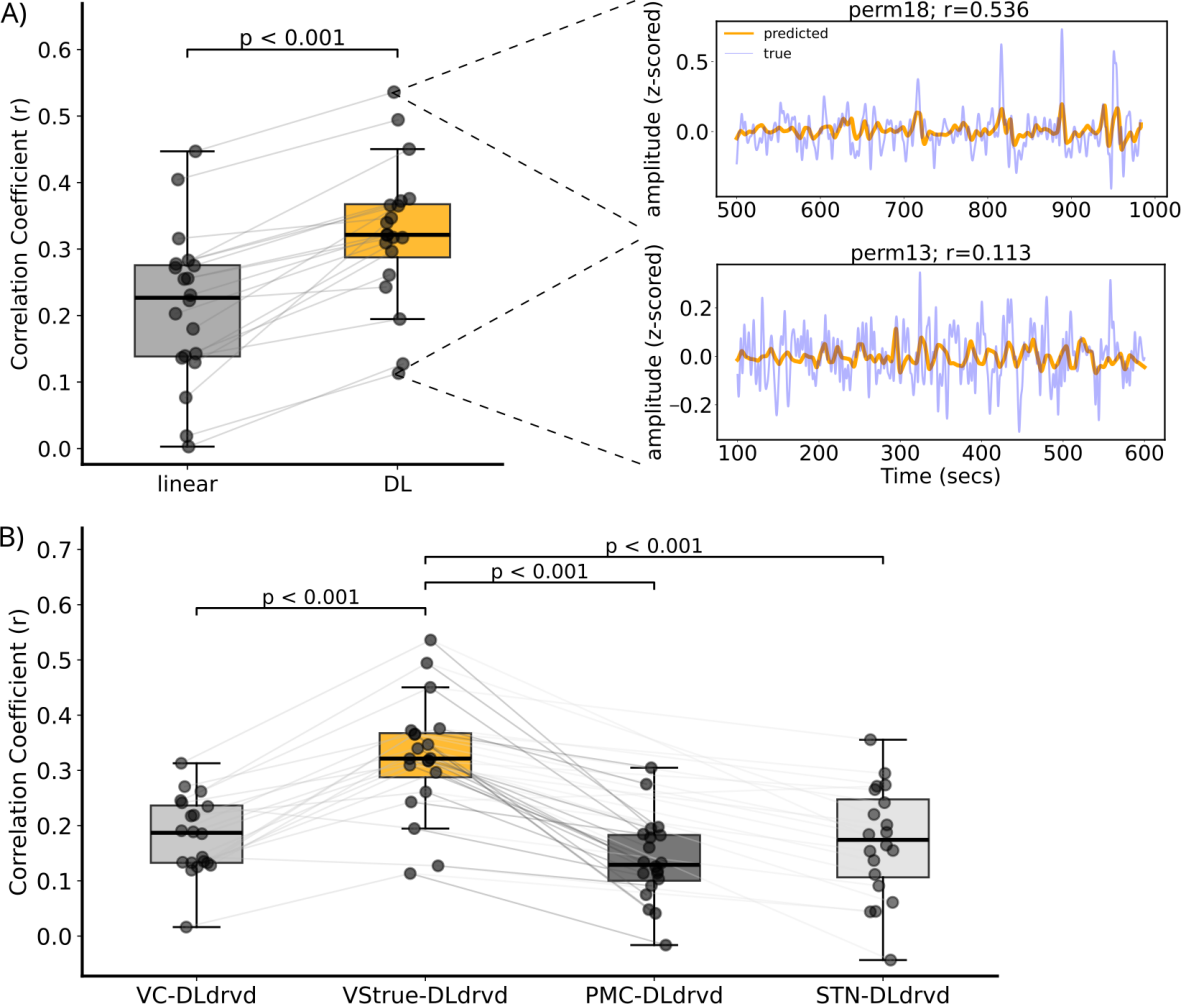
Prediction accuracy of the model for the training dataset. (A) Coefficients for the correlations between the true VS BOLD and the linear model (gray) versus DL-derived (orange) time series. Each datapoint represents one held-out dataset. Example correlations between the DL-derived (orange) and the actual VS BOLD (light blue) signals for the dataset with the highest (r = 0.536, top) and the lowest (r = 0.113, bottom) correlation are depicted on the right. (B) Comparison of correlation coefficients between the DL-derived VS time series (DLdrvd) and control BOLD signals. The orange boxplot replicates the one from panel A. The additional three boxplots show correlations between the same DL-derived VS time series and BOLD signals from three control regions: visual cortex (VC), premotor cortex (PMC), and subthalamic nucleus (STN).

In the present study, we aimed to develop an EEG-based model of brain activity in the VS. To this end, we trained a convolutional autoencoder deep learning model to reconstruct the VS BOLD signal from simultaneously acquired EEG signals. To evaluate the model’s performance, we employed a 20-fold cross-validation procedure and computed the Pearson correlation between the DL-derived time series and the true VS BOLD signal for each held-out dataset. Results show an average correlation of $\bar{r}=0.323$ (*SD* = 0.105, *min* = 0.113, *max* = 0.536). See Figure 3A orange boxplot. The right panel in Figure 3A shows the time series alignment for two example folds: the one with the highest correlation and the one with the lowest correlation. Phase-randomization permutation testing confirmed that 19 of 20 fold-specific correlations were statistically significant (Supplementary Fig. S1). Furthermore, the DL model significantly outperformed the linear baseline model (*t(19)* = 8.280, *p* < .001), which demonstrated an average correlation of $\bar{r}=0.213$ (*SD* = 0.114, *min* = 0.018; *max* = 0.446). See Figure 3A, gray boxplot. To assess whether the model captured VS-specific activity rather than general BOLD dynamics, we compared correlation coefficients between the DL-derived time series and BOLD signals from three control regions: visual cortex (VC), subthalamic nucleus (STN), and premotor cortex (PMC). The DL-derived time series showed a substantially higher correlation with the true BOLD signal from the VS than with the true BOLD signal from any of these control areas (Fig. 3B). Pairwise comparisons revealed highly significant differences for all contrasts: VS versus VC (*t(19)* = 7.803, *p* < .001), VS versus PMC (*t(19)* = 8.762, *p* < .001), and VS versus STN (*t(19)* = 6.742, *p* < .001).

Finally, to address the potential risk of overfitting and inflated accuracy estimates arising from up-sampling the fMRI signal (Simões et al., 2020), we conducted an additional control analysis in which we correlated the true VS BOLD signal sampled at its original 0.5 Hz rate with the DL-derived signal downsampled to the same rate. Although correlations for these downsampled signals were slightly lower (${\bar{r}}_{downsampled}=0.321$, *min_downsampled_* = 0.109, *max_downsampled_* = 0.535) than the correlations from the 100 Hz signals, this reduction was not significant (*p* = .06, *t(19)* = 1.968; see Supplementary Fig. S2).

### 3.2 Anatomical specificity of the model

**Fig. 4.**
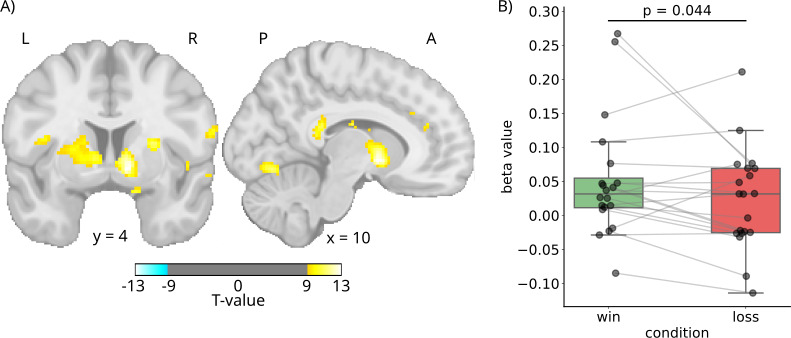
Anatomical specificity and functional validity of the DL-derived time series. (A) Results for the group-level analysis. Using the DL-derived time series as a regressor in a whole-brain random effects fMRI analysis reveals a significant association with the ventral striatum (FWE *p* < .001; peak coordinates: x = 10, y = 4, z = –4; *t(19)* = 13.19). Additionally, the signal correlates with other reward-related regions such as the anterior cingulate cortex, caudate body, putamen, and visual cortex areas. (B) Functional validity of the DL-derived VS signal. The signal exhibits the expected reward modulation, with higher beta values for win (green) than for loss (red) events.

Next, to examine the anatomical specificity of the DL-derived time series, we conducted a whole-brain random-effects general linear model analysis, using the DL-derived time series as a regressor. This analysis revealed that the DL-derived signal significantly predicted BOLD activity within the VS at *p_FWE_* < 0.001 (peak coordinates: x = 10, y = 4, z = –4; *t(19)* = 13.19; Fig. 4A). Beyond this, significant activations were also observed in the anterior cingulate gyrus, caudate body, putamen, and visual association as well as secondary visual cortex. The effect was greatest in the *right* VS, however. Refer to Table 1 for a detailed outline of the coordinates and statistics for all significant clusters.

**Table 1.** Group-level fMRI activation associated with DL-derived time series.

Cluster		Peak					
*p* (FDR-corr)	N voxel	*p* (FWE-corr)	T	x	y	z	Brain area
0	1,497	0	13.19	10	4	-4	right ventral striatum
		0	12.27	18	6	-18	
		0	11.59	-6	0	2	
0	68	0	13.06	66	-18	20	right supramarg. gyrus
		0	10.27	58	-22	20	
0	69	0	12.98	58	-62	8	right visual association cortex
0	174	0	12.71	14	-70	-12	right secondary visual cortex
0	496	0	12.45	-24	-30	20	left caudate body
		0	12.2	-28	-22	22	
		0	11.17	-46	-16	4	
0	190	0	12.02	-40	42	28	left anterior prefrontal cortex
		0	11.79	-32	48	30	
		0.001	9.08	-46	40	20	
0	114	0	11.52	-64	-30	24	left supramarg. gyrus
		0	10.26	-58	-16	18	
		0	9.53	-58	-24	16	
0	288	0	11.29	26	4	12	right anterior putamen
		0	10.26	30	14	12	
		0	10.1	58	8	-10	
0	67	0	11.23	26	46	38	right dorsolateral prefrontal cortex
		0	10.17	34	42	36	
0	166	0	11.2	8	-36	20	right posterior corpus callosum
		0	10.35	-6	-36	14	
		0.001	9.34	12	-36	30	
0.046	19	0	10.86	-16	-42	-14	left fusiform
0	83	0	10.77	-42	-2	14	left supplementary motor cortex
		0	9.47	-38	-8	18	
0	133	0	10.75	62	0	20	right primary motor cortex
		0	9.63	56	12	18	
		0	9.52	50	-4	24	
0	151	0	10.73	34	-16	-6	right posterior putamen
		0	10.17	34	0	0	
		0.001	9.13	30	-8	2	
0.007	35	0	10.72	-10	58	32	left dorsolateral prefrontal cortex
0.025	24	0	10.65	42	56	8	right anterior prefrontal cortex
0.001	59	0	10.64	34	22	26	right dorsolateral prefrontal cortex
		0	10.27	34	20	18	
0	124	0	10.61	40	-82	6	right visual association cortex

### 3.3 Functional validity of the model

Next, to test whether the DL-derived time series is sensitive to reward similarly to the reward-related engagement typically observed in VS BOLD activation, we conducted a subject-level random-effects GLM analysis. The DL-derived time series was treated as the dependent variable, while the HRF modeled reward events (win vs. loss) served as predictors. The results demonstrated that, as expected, the DL-derived signal was reliably modulated by reward conditions, showing higher beta values for win than for loss trials. The difference in betas per condition was significant at *p* = .044; *t(19)* = 1.80. See Figure 4B.

### 3.4 Spatial and temporal EEG characteristics of the model

After the anatomical and reward specificity of the model was confirmed, we next sought to evaluate the spatial and frequency patterns that contributed most to generate the predicted time series. In accordance with Kovalev et al (2022), we use the approach described in Petrosyan et al. (2021). In its essence, this approach uses the learned weights from first point-wise (spatial) and second depth-wise (temporal) 1-D convolutional layers in the interpretable compact block to identify the electrodes and frequency components most critical to the model’s predictions. Out of the 16 available branches, we present results for the 3 branches with the highest influence—that is, those branches that contributed predominantly to the final predicted time series. The reported values reflect the average across folds for the highest, second highest-, and third highest-ranking branch within each fold, respectively.

**Fig. 5.**
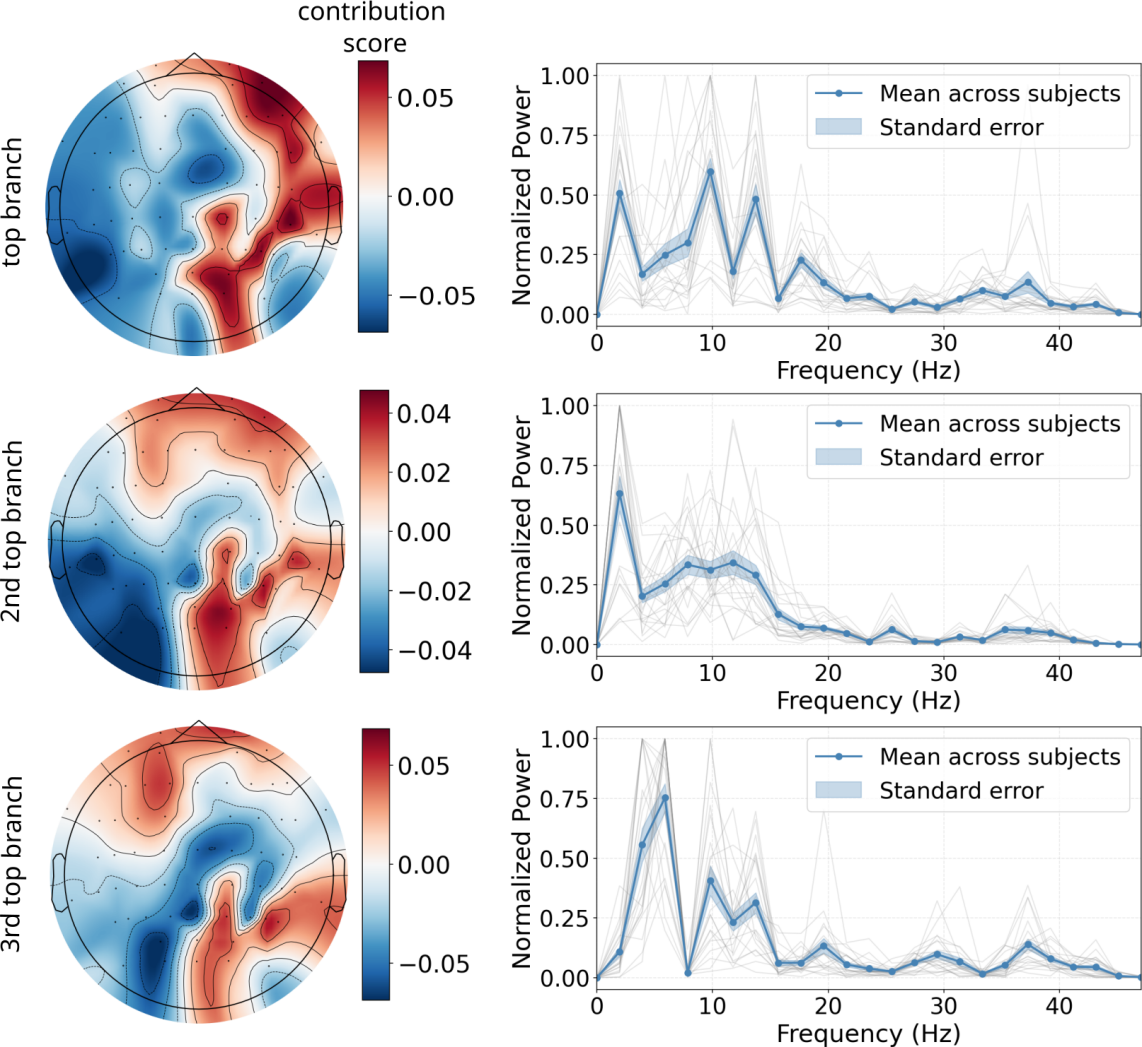
Topoplot and frequency patterns for the top three branches. Left: Electrode contribution scores, averaged across held-out datasets, show widespread activity across the scalp for each of the top three branches. Right: Frequency contributions reveal the involvement of delta, theta-, alpha- and low beta-band activity.

Results show distinct spatial and frequency patterns across the branches (Fig. 5). In the top branch, the highest electrode contributions were observed at right frontotemporal and centroparietal sites. The predominant frequency contribution for this branch occurred at approximately 10 Hz, with a secondary peak at 2 Hz and two more peaks in the low beta range, at around 13 and 18 Hz. In the second top branch, electrode contributions were localized more frontally, with additional involvement of right centroparietal and temporal regions. Frequency analysis for this branch revealed a primary peak contribution of frequencies at around 2 Hz, with a secondary peak near 11 Hz. For the third top branch, the distribution of electrode contributions was even more frontally, with again involvement of centroparietal and temporal regions. This branch demonstrated the most prominent contribution of frequencies at around 6 Hz, with further peaks at 10, 14, and 19 Hz.

## 4 Discussion

The objective of this study was to develop and validate a generalizable EEG probe of the neural activation in the ventral striatum (VS), a core deep brain region integral to the brain’s reward center. Using a deep learning (DL) model, previously developed by Kovalev et al. (2022), we identified a particular spatio-spectral EEG representation that is predictive of a concurrently acquired reward-specific fMRI BOLD time series extracted from the VS. Across a 20-fold cross-validation framework, the model achieved a mean correlation of $\bar{r}=0.323$ between DL-derived and true VS BOLD time series. Furthermore, a series of validation analyses revealed both anatomical specificity and functional sensitivity of the DL-derived signal. Although predictive accuracy remains moderate, the results establish the feasibility of decoding VS-related reward dynamics noninvasively from EEG.

### 4.1 Comparative evaluation of our model

The model demonstrated a moderate predictive performance ($\bar{r}=0.323$), outperforming both our internal linear baseline ($\bar{r}=0.213$) and previously published linear regression-based EEG–fMRI VS models (Singer et al., 2023; $\bar{r}=0.26$ for music reward and $\bar{r}=0.11$ for monetary reward). This improvement is likely due to two factors. First, the use of a deep learning architecture probably allowed the model to learn nonlinear associations between EEG spatio-spectral features and fMRI hemodynamic responses—relationships that may not be adequately modeled using linear regression approaches. Second, compared with Singer et al., the present study utilized substantially more training data. The dataset included simultaneous EEG–fMRI recordings from 19 participants, providing roughly twice the amount of signal used in earlier work. This increased dataset size may have enabled more stable learning of cross-modal structures and thus improved generalization.

Despite outperforming linear baselines, the model’s mean prediction accuracy remained lower than that reported by Kovalev et al. (2022), who achieved a mean validation correlation of $\bar{r}=0.42$ for the nucleus accumbens. A key methodological difference likely contributing to this discrepancy is that Kovalev et al. trained subject-specific models rather than a single group-level model as we do here. This divergence reflects a fundamental trade-off: subject-specific models typically yield higher accuracy because they can capture individual neurophysiological characteristics, such as head or tissue geometry, or electrode positioning. A group-level approach, however, treats individual variability as noise rather than signal, thus constraining predictive precision at the single-subject level. However, the group-level approach presented here enables cross-participant generalizability, a property that is essential for potential clinical applications where individualized fMRI calibration may not be feasible. Importantly, group-level performance typically improves as training datasets grow, reflecting well-described scaling laws in deep learning (Bhatt et al., 2024; Kaplan et al., 2020). Thus, while the present performance does not yet match subject-specific results, the fact that our model achieved meaningful generalization across participants suggests that the chosen approach is viable and that increasing training cohort size may further close this gap in future work.

### 4.2 Anatomical specificity and functional validity

Whole-brain regression analysis supported the anatomical specificity of the DL-derived time series, showing significant associations with the VS. Additionally, we observed associations in regions known to be functionally coupled to the VS, such as the anterior cingulate cortex, caudate, putamen, PFC, and visual cortex areas (Haber & Knutson, 2010). Importantly, this pattern closely resembles that of the actual VS BOLD signal (see Supplementary Fig. S3A), and generally aligns with established patterns of VS network connectivity (e.g., Andreou et al., 2017; Haber, 2011; Hartmann et al., 2023; Herzog et al., 2024b). In addition, our anatomical findings are remarkably similar to those presented in Singer et al. (2023).

When comparing correlation coefficients between the DL-derived VS time series and the true BOLD signals from control regions (visual cortex, premotor cortex, and subthalamic nucleus), we found that the predicted VS time series was significantly stronger correlated with the true VS signal than with the BOLD signal from any of these control areas. At the same time, moderate correlations with the control regions were observed, which was especially true for the visual cortex. This finding is not entirely unexpected, as the visual cortex was also implicated in our anatomical sensitivity analysis. Moreover, this finding likely reflects task-specific functional coupling, given that our paradigm relied on visual stimuli to signal reward cues. This interpretation is supported by prior evidence that visual input can influence reward-based decision making and VS activity (Cicmil et al., 2015; Takagaki & Krug, 2020). Although our rationale for selecting these specific control regions was their lack of canonical involvement in the reward circuit, the observed moderate correlations for each control region may nonetheless reflect indirect but meaningful functional coupling. Alternatively, or in addition, these correlations may represent a baseline level of similarity between each two time series data due to shared power-law dynamics (see Schaworonkow et al., 2015). Either way, the fact that correlations with the VS BOLD signal were significantly stronger provides compelling evidence that the model’s predictions reflect region-specific neural dynamics rather than global BOLD signal properties.

Functionally, the DL-derived VS signal was modulated by reward conditions, with higher beta values for win than for loss events. This differential response pattern mirrors the canonical BOLD response profile seen in our ground truth VS signal (Supplementary Fig. S3B), as well as general patterns of VS activation during reward processing reported in the literature (e.g., Grill et al., 2021; Knutson & Greer, 2008). However, the difference in beta values between win and loss conditions was less pronounced for the DL-derived signal compared with the ground truth VS BOLD signal. This attenuation of the reward response magnitude emphasizes that, while the model captures the general direction of reward-related modulation, further refinement of the model is needed to fully recover the sensitivity of the native BOLD signal. Nevertheless, the fact that the DL-derived signal shows functionally appropriate modulation already validates the model as a promising foundation for an EEG-based proxy of VS engagement.

### 4.3 Spatial and temporal EEG characteristics of the model

To better understand which EEG features the model relied on, we applied the approach described in Petrosyan et al. (2021) to extract the respective spatial and frequency features. For all top three branches (i.e., the model’s strongest spatio-spectral processing streams), results revealed rather widespread topographical contributions across the scalp. If we interpret the contribution scores to be dipoles, these findings suggest the involvement of deep neural sources, as electrical activity originating from deeper brain regions tends to produce more diffuse and less focal scalp patterns due to volume conduction and spatial blurring (Hnazaee et al., 2020; Unnwongse et al., 2023).

Notably, all branches showed prominent involvement of right temporal electrodes—a spatial distribution that closely resembles the topographies reported by Singer et al. (2023; see their Supplementary Fig. S1) and aligns with the temporal lobe associations observed in our whole-brain regression analysis (Fig. 3B). Additionally, the centroparietal pattern is consistent with that reported in Kovalev et al. (2022) for nucleus accumbens predictions. The right-hemispheric bias in the scalp features furthermore aligns with our fMRI results showing strongest associations in the right VS, and converges with prior work indicating right-lateralized engagement of striatal circuits during reward processing (e.g., Andreou et al., 2017). Notably, this lateralization may be further explained by our exclusively male sample, given that sex differences in reward-system asymmetry have been documented (Martin-Soelch et al., 2011). Finally, also the frontal contributions observed in the branches are consistent with our anatomical specificity analysis, potentially reflecting the involvement of ACC and/or PFC.

In terms of spectral contributions, the model relied most strongly on delta, theta, alpha, and low beta frequency bands. These findings align well with prior findings. For instance, using the same dataset, Andreou et al. (2017) identified mid-frontal theta and beta oscillations as markers of loss and win outcomes, respectively. Similarly, Singer et al. (2023) report alpha- and beta-band contributions for their EEG-derived VS model, and Kovalev et al. (2022) likewise find that their subject-specific models relied on these frequencies. More broadly, these spectral dynamics have been documented in various reward paradigms (Andreou et al., 2015; Chen & Kwak, 2022; Cohen et al., 2011). Across these studies, theta-band oscillations have been linked to negative feedback processing, while beta-band activity was more commonly associated with positive feedback processing. Beyond feedback valence, beta oscillations have also been associated with motor preparation and anticipatory processing in reward contexts (Bunzeck et al., 2011; Doñamayor et al., 2012), and link to dopaminergic signaling (Gerster et al., 2025; Jenkinson & Brown, 2011; Oswal et al., 2013). While delta oscillations are less commonly highlighted in reward paradigms, their presence may reflect dynamic recruitment of parallel neural pathways, as delta oscillations are also known to synchronize distant brain regions (e.g., Nácher et al., 2013).

Overall, our model appears to successfully capture multiple aspects of the EEG correlates of reward processing, integrating both spatial and spectral features consistent with the literature

### 4.4 Implications of our findings

The overall goal of this study was to develop a generalizable EEG-based model of VS reward signaling that may ultimately be suitable for clinical application. Inter-subject generalizability is essential for this purpose, and our findings demonstrate that such generalization is at least partially achievable. Notably, our mean validation correlation exceeds those reported by Singer et al. (2023), who obtained $\bar{r}=0.26$ in their primary dataset and $\bar{r}=0.11$ during external validation. Despite these modest values, their model demonstrated clinical utility (Amital et al., 2025), suggesting that absolute correlation strength may not be the sole determinant of translational value. This indicates that our approach, even at its current performance level, may as well already hold clinical relevance. However, increasing dataset size and diversity for training the model will likely further strengthen the model’s specificity, and thus its value for clinical use. Future studies should empirically test this assumption.

### 4.5 Limitations and future directions

Despite our promising findings, several limitations should be acknowledged. First, the model was trained and evaluated using data from male participants only, limiting its applicability to broader, more diverse populations. Improving generalizability for a broader population will require training on larger and more heterogeneous datasets. Additionally, research has shown that functional and neuroanatomical characteristics of the VS and its associated reward-network regions can vary across different psychiatric conditions (Der-Avakian & Markou, 2012; Pizzagalli, 2014; Waltmann et al., 2024) or with age (Scholz et al., 2023, 2024; Schreuders et al., 2018; Vink et al., 2015). Therefore, future work should aim to develop models that can account for these variations across different sub-populations. For studies focusing on personalized precision rather than scalability, it may be beneficial to train individualized rather than group models.

Furthermore, our model may have over-fitted to task-specific VS dynamics, as it was trained on data from one specific reward task only. It would hence be valuable to perform an out-of-sample validation where the model’s performance is evaluated across different reward domains, such as primary (e.g., food) or secondary (e.g., music) rewards. Such tests would help determine whether the model captures general reward mechanisms or is task dependent, thus determining its translational impact.

Additionally, our permutation-based significance testing used phase-randomized BOLD signals and correlates these surrogate signals with the DL model predictions. A more rigorous approach would involve training models on phase-shifted data and correlates their predictions with true BOLD signals (Mehta et al., 2025). While we acknowledge this, implementing such an approach was computationally prohibitive given the need to retrain numerous surrogate models. Nonetheless, our approach is consistent with prior published work (Singer et al., 2023) and the overall pattern of results provides converging evidence for the validity of the model.

Next, while the DL-derived time series showed strong associations with the VS, it also correlated with several cortical regions known to be part of the broader reward network. While this complicates strict anatomical interpretation, it may not pose a practical limitation, however. Similar network-wide patterns were observed for the amygdala and VS EEG models from Meir-Hasson et al. (2014) and Singer et al. (2023), respectively. Yet, their models were clinically effective (Amital et al., 2025; Meir-Hasson et al., 2016).

Finally, we used a fixed 6-second BOLD delay when training the model, but there is evidence that optimal HRF timing may vary depending on frequency band, dataset, or the underlying networks involved (Xavier et al., 2025). Future work could incorporate an additional convolutional layer within the compact block to learn individual delay shifts dynamically, which may improve precision and alignment with BOLD signals.

### 4.6 Final conclusions

Overall, this study contributes to the emerging field of EEG-based decoding of neural activity from deep brain structures (e.g., Attal et al., 2012; Michel & Brunet, 2019; Pizzo et al., 2019) and provides support for the feasibility of using deep learning to infer VS activity from EEG recordings. While inter-subject generalization remains an ongoing challenge, the current findings represent a meaningful step toward practical, EEG-based tools for monitoring subcortical reward activity. Such tools could, for example, support the development of EEG-based neurofeedback interventions for disorders characterized by dysfunctional reward processing, such as depression (Admon & Pizzagalli, 2015; Pizzagalli, 2014), borderline personality disorder (Andreou et al., 2015; Schauer et al., 2021), or substance use disorders (Solinas et al., 2019; Wise & Robble, 2020).

## Supplementary Material

Supplementary Material

## Data Availability

The pre-processed data, the codes used to train and evaluate the model, and the final weights are available here: https://github.com/naherzog/DLEEGfMRI. Requests for raw EEG or fMRI data will be evaluated individually and must comply with local regulations and the requirements of German authorities.
