# Supplementary material for: From surface to depth: Using deep learning to predict striatal fMRI reward signaling from EEG
Source: Imaging Neurosci (Camb). 2026 Mar 9;4:IMAG.a.1160. doi: 10.1162/IMAG.a.1160 (PMC12973074; doi:10.1162/IMAG.a.1160)
Supplement: Supplementary Material [file IMAG.a.1160_supp.pdf]

SUPPLEMENTS

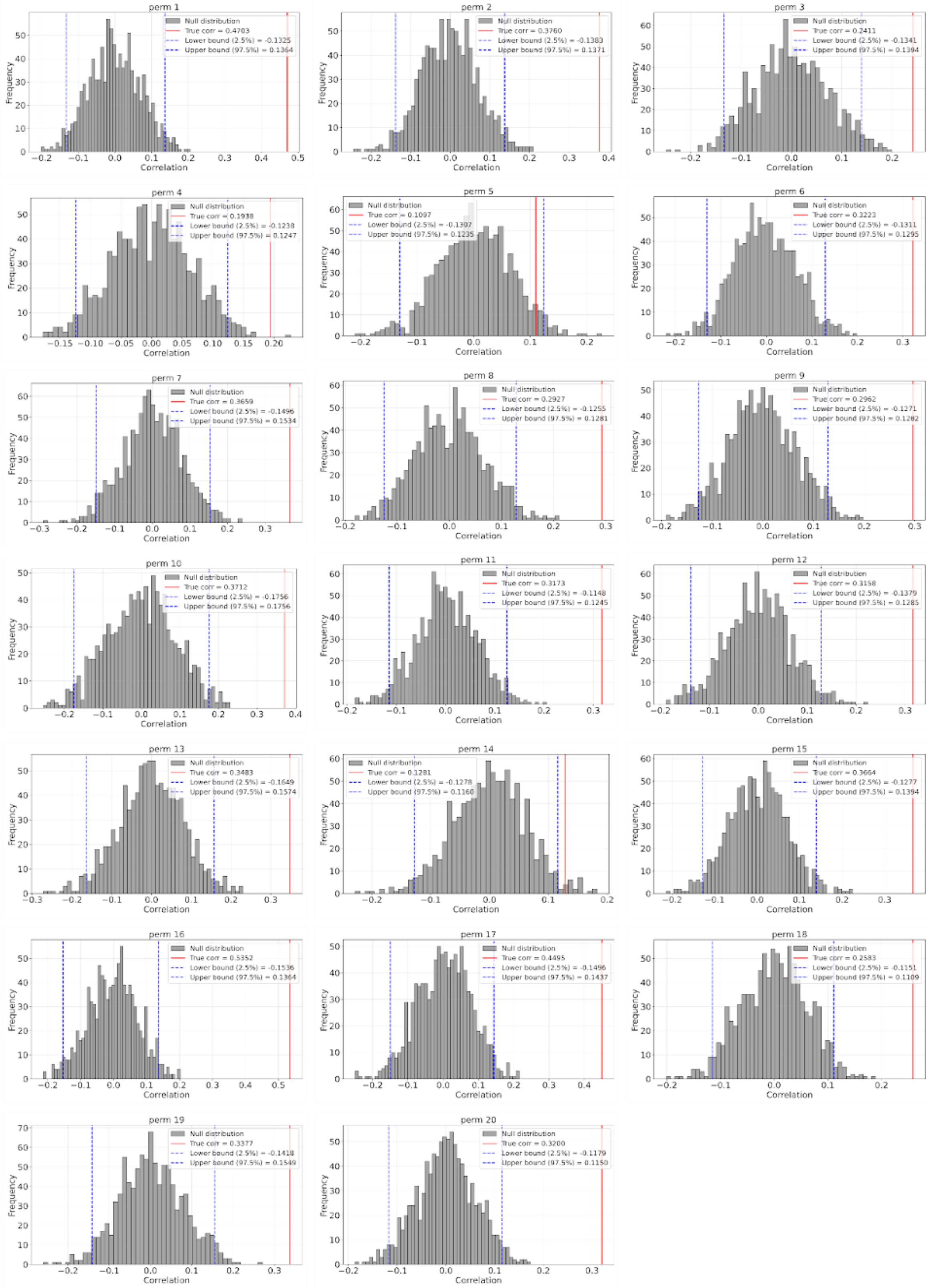

Figure S1. Results for the phase-shifted permutations per fold. All but one (perm5) empirical correlation values were significant.

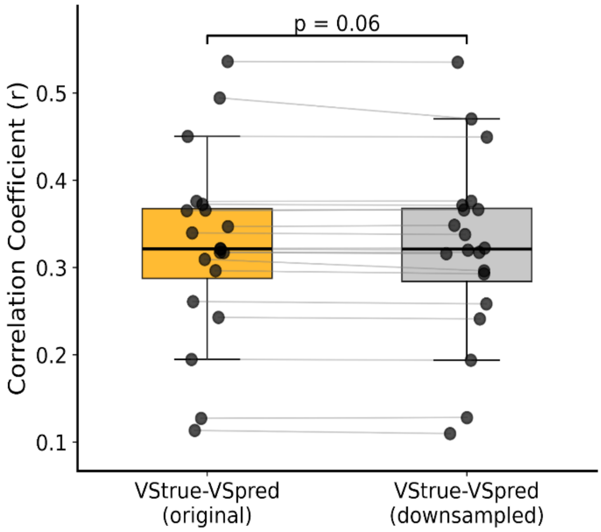

Figure S2. Control analysis for the DL-derived vs. true VS signals at the original, for training used sampling rate (100Hz) and at a downsampled rate (0.5 Hz).

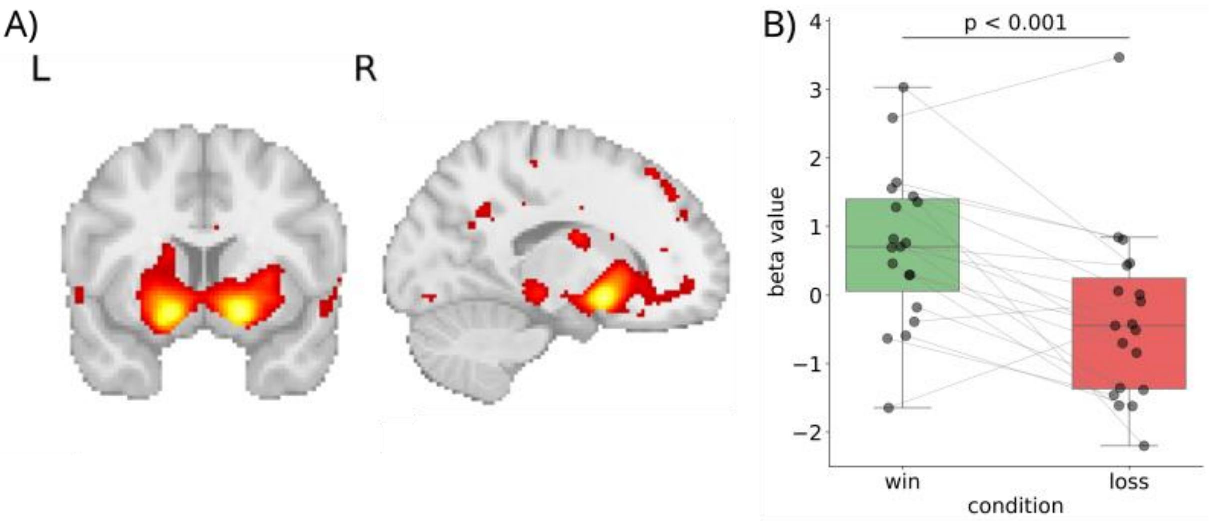

Figure S3. A) Anatomical correlations of the ground truth VS BOLD signal. **B)** Beta values per fold for win and loss events for the true VS BOLD signal.
